# Perspectives on menstrual policymaking and community-based actions in Catalonia (Spain): a qualitative study

**DOI:** 10.1186/s12978-023-01730-9

**Published:** 2024-01-04

**Authors:** Andrea García-Egea, Anna Sofie Holst, Constanza Jacques-Aviñó, Cristina Martínez-Bueno, Anna Berenguera, María Mercedes Vicente-Hernández, Carme Valls-Llobet, Diana Pinzón-Sanabria, Georgina Pujolar-Díaz, Laura Medina-Perucha

**Affiliations:** 1 Fundació Institut Universitari per a la Recerca a l’Atenció Primària de Salut Jordi Gol i Gurina (IDIAPJGol), Gran Via de les Corts Catalanes 587 attic, 08007 Barcelona, Spain; 2 Universitat Autònoma de Barcelona, Bellaterra, Cerdanyola del Vallès Spain; 3 Network for Research on Chronicity, Primary Care, and Health Promotion (RICAPPS), Barcelona, Spain; 4 Servei d’Atenció a la Salut Sexual i Reproductiva (ASSIR), Direcció Assistencial d’Atenció Primària, Institut Català de la Salut, Barcelona, Spain; 5 Sexual and Reproductive Health Care Research Group (GRASSIR), Barcelona, Spain; 6 Universitat de Barcelona, Barcelona, Spain; 7 Departament d’Infermeria, Universitat de Girona, Girona, Spain; 8 Centro de Análisis y Programas Sanitarios (CAPS), Barcelona, Spain; 9 SomiArte Taller, Manresa, Spain

**Keywords:** Menstrual inequity, Menstrual health, Menstrual cycle, Menstruation, Women’s Health, Gender-based research, Menstrual policymaking, Qualitative research, Photoelicitation, World Café, Inequidad menstrual, Salud Menstrual, Ciclo menstrual, Menstruación, Salud de las mujeres, Investigación de género, Legislación menstrual, Metodología cualitativa, Fotoelicitación, World Café

## Abstract

**Background:**

Menstrual research and policymaking have become imperative worldwide. It is necessary that these are informed by women and people who menstruate (PWM) alongside expert professionals and activists.

**Methods:**

The main aim of this study was to identify and propose policies and community-based actions to address menstrual inequity and promote menstrual health in Catalonia (Spain). This study consisted of two qualitative studies: (a) 34 individual photoelicitation interviews with women and PWM, (b) a World Café study with 22 professionals and activists. Sampling for both studies was purposive and selective. Recruitment was conducted through healthcare centres, social media, key contacts, and snowball sampling techniques. Data were collected in December 2020-September 2022, and analysed using Framework Analysis.

**Results:**

Participants considered the implementation of menstrual policies that address the taboo and stigma of menstruation to be crucial. They stressed the need for menstrual education, which should be integrated into formal education curricula. Participants, and especially women and PWM, highlighted the need to improve the access and quality of healthcare services, so that the menstrual cycle and menstruation are seen as health indicators. Health professionals should encourage agentic informed decisions, hence why both participant groups considered menstrual health education amongst health professionals to be pivotal. Taking action to improve the access and affordability of menstrual products was also imperative for participants, especially for socioeconomically vulnerable populations. Participants agreed on guaranteeing fully equipped menstrual management facilities, and and professionals discussed gender-neutral and sex-segregated bathrooms. Workplace menstrual policies to accommodate and ensure menstrual self-care were also suggested.

**Conclusions:**

Our study highlights the need for multi-dimensional menstrual policies. These should include actions to address menstrual taboo and stigma, to promote menstrual education that goes beyond the hegemonic biomedical prism, to improve the access and quality of menstrual health services, along with policies ensuring adequate menstrual management facilities in public spaces and the access to menstrual products. Policymaking should also focus on how to ensure menstrual management and care in workplaces. Menstrual policies and community-based actions should be framed within intersectionality, to consider how societal structures of power and oppression influence menstrual experiences.

**Supplementary Information:**

The online version contains supplementary material available at 10.1186/s12978-023-01730-9.

## Plain language summary

Research has shown the growing importance of understanding and creating policies related to menstruation worldwide. In Catalonia, Spain, a study was conducted involving both women and individuals who menstruate, as well as experts and activists to uncover the best ways to address menstrual-related issues. Through interviews and group discussions, participants shared their thoughts on how to improve menstrual health and reduce inequities. They highlighted the urgency of tackling societal taboos surrounding menstruation and incorporating menstrual education into school curricula. There was a call for better healthcare services that view menstrual health as an indicator of overall health. Participants felt strongly about improving access to menstrual products, especially for those with financial difficulties. There was also consensus on the need for well-equipped public facilities to manage menstruation, including discussions on gender-neutral and separate bathrooms. Suggestions were made to have policies in workplaces that support individuals during their menstruation. The study concludes that comprehensive policies are needed to address a range of menstrual concerns, from education to public facilities, with an emphasis on understanding the diverse experiences and challenges individuals face due to societal structures.

**Supplementary Information:**

The online version contains supplementary material available at 10.1186/s12978-023-01730-9.

## Resumen

**Introducción:**

Siendo la investigación y la implementación de políticas menstruales imprescindibles, es necesario que estos procesos estén informados por mujeres y personas que menstrúan (PM), así como por profesionales expertas y activistas.

**Métodos:**

El objetivo principal de este estudio fue identificar y proponer políticas y acciones comunitarias para abordar la inequidad menstrual y promover la salud menstrual en Cataluña (España). Este estudio consistió en dos estudios cualitativos: (a) 34 entrevistas individuales de fotoelicitación con mujeres y PM, (b) un World Café con 22 profesionales y activistas. El muestreo para ambos estudios fue intencional y selectivo. El reclutamiento se realizó a través de centros de salud, redes sociales, contactos clave y técnicas de bola de nieve. Los datos se recogieron entre diciembre de 2020 y septiembre de 2022 y se analizaron mediante Framework Analysis.

**Resultados:**

Las participantes consideraron crucial la implementación de políticas menstruales para abordar el tabú y el estigma menstrual. Destacaron la necesidad de una educación menstrual, que debería integrarse en los currículums escolares. Las participantes, y especialmente las mujeres y PM, resaltaron la necesidad de mejorar el acceso y la calidad de los servicios de salud, de manera que el ciclo menstrual y la menstruación sean consideradas indicadores de salud. Mencionaron que el personal sanitario debe fomentar las decisiones informadas, de ahí que ambos grupos de participantes consideraran fundamental la educación sobre la salud menstrual entre los profesionales de la salud. También, para las participantes fue imperativo asegurar el acceso y asequibilidad de productos menstruales, especialmente para las poblaciones socioeconómicamente vulnerabilizadas. Las participantes estuvieron de acuerdo en la necesidad de garantizar espacios equipadas para el manejo menstrual, y se llevaron a cabo debates entre las profesionales sobre los baños inclusivos y segregados por sexo. También se sugirieron y debatieron políticas menstruales en entornos laborales, para adaptar y garantizar el autocuidado menstrual.

**Conclusiones:**

Nuestro estudio destaca la necesidad de políticas menstruales multidimensionales. Estas deberían incluir acciones para abordar el tabú y el estigma menstrual, promover una educación menstrual que vaya más allá de la perspectiva biomédica hegemónica, mejorar el acceso y la calidad de los servicios de salud menstrual, junto con políticas para garantizar la disponibilidad de instalaciones adecuadas para el manejo menstrual en espacios públicos, así como el acceso a productos menstruales. La creación de políticas también debería centrarse en cómo garantizar el manejo y los cuidados menstruales en entornos laborales. Finalmente, estas políticas menstruales y acciones comunitarias deben enmarcarse desde la interseccionalidad, para considerar cómo las estructuras y poderes sociales operan e influyen en las experiencias menstruales.

## Background

Menstrual justice [1, 2] is based on the understanding that menstrual processes occur within a socioeconomic context. It highlights menstrual discrimination, inequalities/inequities and injustice. In doing so, it directs the attention towards the socio-political patriarchal structures in which women and people who menstruate (PWM) experience menstrual oppressions. Further, Margaret Johnson proposes that menstrual justice should be embedded within a structural intersectionality lens [2, 3]. Our research has particularly focused on *menstrual inequities* [4] as the systematic and avoidable disparities in the access to menstrual healthcare, education, products and services to healthily manage menstruation, the experiences of menstrual stigma and discrimination, and the barriers to social, economic and political participation based on having a menstrual cycle and menstruating [5, 6]. For instance, a recent study in Spain has indicated that self-reported lifetime menstrual poverty affects 22.2–39.9% of women and PWM, especially those who experience financial hardship or do not have a permit to reside in Spain [6]. This framework sets its grounds on understanding and addressing menstrual inequities experienced by more vulnerabilised and discriminated communities.

Activism has successfully taken the lead in placing menstruation in the public eye and on policy agendas worldwide [4, 7, 8]. A recent study, which combines a review of menstrual policies in India, Kenya, Senegal and the United States and key informant interviews, suggests that menstrual policymaking is largely focused on tangible and material outcomes [9]. Besides, as the authors indicate, there is a tendency for policymaking to limit menstrual policy to menstrual management. Thus, most policies focus on provision of menstrual products (particularly in schools) and menstrual facilities. This can be concerningly short-sighted to other menstrual needs. It operates alongside the minimisation and negation of the need to ensure menstrual equity and health, rooted in gender inequity and discrimination [9].

Policy discussions in Spain were especially initiated since the start of the COVID-19 pandemic, and particularly driven by menstrual activism. In 2021, policymakers in Spain made legislative proposals that mirrored other policies in Europe to “eradicate” menstrual poverty [10]—often defined as relating to the financial barriers to access menstrual products—and implement menstrual leaves. Alongside a tax reduction to menstrual products, the Spanish Government approved a menstrual policy in early 2023 (Ley Orgánica 1/2023) [11]. This policy recognises the rights to menstrual education and health, menstrual leaves for secondary dysmenorrhea, and aims to ensure the access to menstrual products for all women and PWM across Spain. In parallel, the Catalan government started a menstrual equity intervention for secondary education students in 2022, to promote menstrual education and healthy menstrual management. However, these policies were barely informed by research in our context and were criticised for undermining their potential backlash on the health, wellbeing, and rights of women and PWM. These measures do not seem to consider the complexity of menstrual inequities, and may fall into becoming reductionist, tokenistic and ineffective actions. As such, tax reduction on menstrual products cannot become a stand-alone policy to “eradicate” or even address menstrual poverty. Moreover, special attention needs to be paid so that policies in Spain are inclusive of all populations who menstruate and avoid the exacerbation of inequities, as it has been the experience in other countries [12, 13]. Besides, the 1/2023 policy does not recognise menstrual leave rights for women and PWM with primary dysmenorrhea and for those with informal employment or dedicated to social reproduction. The question also remains on how these policies will be translated into institutional actions.

The main aim of this study was to identify and propose policies and community-based actions to address menstrual inequity and promote menstrual health in Catalonia (Spain), based on both the experiences of women and PWM and professionals and activists in health, education, social and politics/public administration sectors. This research could be useful to inform policymakers into what directions to take when trying to address menstrual inequities in our context.

## Methods

The study is embedded in a larger mixed-methods project, the “Equity and Menstrual Health in Spain study” (2020–2022) [6, 14], which has focused on identifying and exploring experiences of menstrual equity and health among women and PWM in Spain. A critical feminist perspective is foundational to this research, to critically analyse how societal systemic and structural powers have an impact on health [15–17]. The feminist approach signifies taking a social equity lens to conduct research that supports collective freedom, justice, and equity. A situated knowledge perspective [18] and the practice of reflexivity have been central to our work. The research has been led by a group of women researchers that question the structural invisibilisation of menstrual experiences and who are committed to investigate menstrual experiences from a health equity perspective.

This study encompasses two qualitative studies. For the first study, individual photoelicitation interviews were conducted with women and PWM. The second study consisted of a World Café session with professionals and activists in health, education, social and politics/public administration. Further details on the methodology are provided below.

The quality of this research has been ensured by following the Yardley criteria [19]. The Critical Appraisal Skills Programme (CASP) quality checklist [20] has been used and can be found in Additional file 1: Material S1.

### Study 1. Photoelicitation interviews

#### Participants

Participants were women and PWM aged 18 and older, who had active menstruations and living in the Barcelona area. Exclusion criteria included having entered menopause or not having menstruated for over a year. See Table 1 for more details.

**Table 1:** Participant characteristics: women and people who menstruate (N = 34)

Participant ID	Age	Gender	Identifies as trans	Country of birth	Administrative status	Completed education	Employment status
P_WPWM_1	27	Woman	No	Spain	Spanish nationality	Primary education	No employment/income
P_WPWM_2	40	Woman	No	Spain	Spanish nationality	Secondary education	Employed full-time
P_WPWM_3	23	Woman	No	Spain	Spanish nationality	Professional education	Maternity leave
P_WPWM_4	24	Woman	No	Spain	Spanish nationality	University studies	Employed full-time
P_WPWM_5	25	Woman	No	Spain	Spanish nationality	University studies	Employed full-time
P_WPWM_6	29	Not sure	Not sure	Spain	Spanish nationality	University studies	Self-employed
P_WPWM_7	33	Woman	No	Spain	Spanish nationality	University studies	Employed full-time
P_WPWM_8	35	Woman	No	Spain	Spanish nationality	University studies	Employed full-time
P_WPWM_9	24	Woman	No	Spain	Spanish nationality	University studies	Employed full-time
P_WPWM_10	33	Woman	No	Spain	Spanish nationality	University studies	Employed full-time
P_WPWM_11	33	Woman	No	Spain	Spanish nationality	University studies	Employed full-time
P_WPWM_12	25	Woman	No	Spain	Spanish nationality	University studies	Employed full-time; Studies part-time
P_WPWM_13	25	Woman	No	Spain	Spanish nationality	University studies	Employed full-time
P_WPWM_14	26	Woman	No	Spain	Spanish nationality	University studies	Studies full-time
P_WPWM_15	25	Woman	No	Spain	Spanish nationality	University studies	Employed full-time
P_WPWM_16	47	Woman	No	Spain	Spanish nationality	Professional education	Employed full-time
P_WPWM_17	34	Woman	No	Spain	Spanish nationality	University education	Employed full-time
P_WPWM_18	23	Woman and non-binary	Not sure	Spain	Spanish nationality	Professional education	Medical leave; Studies part-time
P_WPWM_19	25	Woman	No	Spain	Spanish nationality	Secondary education	Employed part-time; Studies full-time
P_WPWM_20	20	Woman	No	Spain	Spanish nationality	Secondary education	Studies full-time
P_WPWM_21	35	Woman	No	Spain	Spanish nationality	University studies	Self-employed; Studies part-time; Unpaid care work
P_WPWM_22	18	Woman	No	Spain	Spanish nationality	Secondary education	Studies full-time; Unpaid care work
P_WPWM_23	28	Woman	No	Spain	Spanish nationality	University education	Employed full-time
P_WPWM_24	20	Non-binary	Yes	Spain	Spanish nationality	Secondary education	Studies full-time
P_WPWM_25	37	Woman	No	Morocco	Permanent residence	Professional education	Employed full-time
P_WPWM_26	24	Woman	No	Spain	Spanish nationality	Professional education	Employed part-time
P_WPWM_27	35	Woman	No	Columbia	Spanish nationality	University studies	Unemployed
P_WPWM_28	37	Woman	No	Spain	Spanish nationality	University studies	Employed full-time
P_WPWM_29	23	Woman	No	Argentina	Refugee status	Secondary education	No income
P_WPWM_30	22	Woman	No	Spain	Permanent residence	Professional education	Employed full-time
P_WPWM_31	25	Woman	No	Pakistan	Permanent residence	Professional education	Employed full-time
P_WPWM_32	29	Woman	No	Spain	Spanish residence	University studies	Employed full-time
P_WPWM_33	28	Woman	No	Spain	Spanish nationality	University studies	Employed full-time
P_WPWM_34	38	Woman	No	Brazil	Spanish nationality	Professional education	Unemployed

*ASSIR = sexual and reproductive healthcare centre

#### Sampling and recruitment

Sampling was purposive and selective. Participants were recruited through social media and public sexual and reproductive health care centres. Snowball sampling techniques were also applied.

#### Data collection

Semi-structured photoelicitation individual interviews were conducted with 34 women and PWM between December 2020 and February 2021. Using photoelicitation was particularly useful to elicit emotional responses and explore taboo topics [21, 22]. This technique facilitates interview processes, especially when discussing sensitive topics (e.g., menstruation). The use of images can reinforce themes that are verbally discussed and can help elicitate emotional responses, memories, attitudes and prejudices, that are otherwise difficult to emerge in a merely conversational interview. A topic guide, devised by the research team, was used to guide the interviews (see Additional file 1: Material S2). Two photographs were presented during the interview, one showing a marathon runner with visible menstrual blood and another with demonstrations against the taxation on menstrual products (see Additional file 1: Material S3, Figs. S1 and S2). This article includes data associated with the aim of this paper only. The interviews were conducted face-to-face (N = 11) either outside in public spaces (such as parks and public squares) or in sexual and reproductive health centres. Other interviews were also conducted over the phone (N = 23) due to COVID-19 restrictions at the time of data collection. The duration of the interviews was between 40 and 85 min and were conducted by LMP and ASH. As a token of thanks, participants received a multi-journey travel card.

### Study 2. World Café

#### Participants

A total of 22 professionals, aged between 26 and 66, participated in the study. All identified their gender as “woman”; one participant indicated her sex (instead of gender). All participants except for one were born in Spain; 20 had completed university education, and most were employed full time at the time of data collection. Participants worked in the health (N = 11), education (N = 5), politics/public administration (N = 4), and social/activism (N = 2) sectors in Catalonia. They had between 1 and 35 years of experience working on menstrual health and equity. See Table 2 for further details.

**Table 2:** Participant characteristics: professionals and activists in menstrual health and equity (N = 22)

Participant ID	Age	Gender	Country of birth	Completed education	Employment situation	Employment occupation	Area of experience	Years of experience
P_PA_1	34	Woman	Spain	Secondary education	Employed full-time and self-employed	Member of parliament and writer	Politics/public administration	4 years
P_PA_2	50	Woman	Spain	University education	Employed full-time	Paediatric nurse	Health	25 years
P_PA_3	46	Woman	Spain	University education	Employed full-time	Civil servant public administration (Equality and Feminism)	Politics/public administration	3.5 years
P_PA_4	48	Woman	Spain	University education	Employed full-time	Nurse	Health	20 years
P_PA_5	53	Woman	Spain	University education	Employed full-time	Midwife	Health	22 years
P_PA_6	36	Woman	Spain	University education	Employed full-time	Special needs teacher	Education	7 years
P_PA_7	40	Woman	Spain	University education	Employed full-time	Midwife	Health	10 years
P_PA_8	31	Woman	Spain	University education	Employed full-time	Civil servant public administration (Equality and Feminism)	Politics/public administration	3 years
P_PA_9	50	Woman	Uruguay	Secondary education	Retired/Health benefits	Activist	Social/Activism	Unknown
P_PA_10	52	Woman	Spain	University education	Employed full-time	Primary healthcare nurse	Health	27 years
P_PA_11	35	Woman	Spain	University education	Employed full-time	Midwife	Health	8 years
P_PA_12	66	Woman	Spain	University education	Employed full-time	Gynaecologist/Sexologist	Health	43 years
P_PA_13	44	Woman	Spain	University education	Employed full-time	Sex educator	Education	6 years
P_PA_14	33	Woman	Spain	University education	Employed full-time	Midwife	Health	8 years
P_PA_15	41	Woman	Spain	University education	Employed full-time	Midwife	Health	18 years
P_PA_16	36	Woman	Spain	University education	Employed full-time	University professor	Education	14 years
P_PA_17	39	Woman	Spain	University education	Employed part-time	Secondary education teacher and menstrual educator	Education	12 years
P_PA_18	38	Woman	Spain	University education	Employed full-time	Midwife	Health	10 years
P_PA_19	26	Woman	Spain	University education	Employed full-time	Sex educator	Education	1 year
P_PA_20	59	Woman	Spain	University education	Employed full-time	Civil servant public administration (Equality and Feminism)	Politics/Public administration	35 years
P_PA_21	50	Woman	Spain	University education	Employed full-time	Midwife/Sexual and reproductive health centre coordinator	Health	17 years
P_PA_22	59	Woman*	Spain	University education	Employed part-time	Psychologist/Employment advisor and educator	Social/Activism	25 years

*Note that P22 indicated her “sex” instead of “gender”

#### Sampling and recruitment

Sampling was purposive and selective. Participants were recruited through key contacts and snowball techniques, based on their experience working on menstrual health and equity. Discursive diversity was ensured by recruiting professionals from different areas (health, education, social, politics/public administration, and social/activism). The research team sent invitation emails to 36 potential participants and 24 accepted to take part; 22 attended the session.

#### Data collection

The World Café is a participatory technique that aims to foster collective and creative discussions in a group of individuals around a given topic [23]. It is particularly useful to define priorities and policy/research recommendations and strengthen and build community networks [24–26]. This technique was considered appropriate to reach the aim set for this study, as it could support identifying policy and community-based actions in a cocreative space.

One World Café session was conducted on the 28th of September 2022, by three experienced qualitative researchers (CJA, CMB, LMP), supported by AGE. The session was three hours long and was audio-recorded for analytical purposes. It took place at the Institut Català de la Salut (Catalan Health Institute) in Barcelona.

First, participants were welcomed and divided into three working groups, which were set in separate rooms and led by CJA, CMB and LMP. Each group was composed by eight participants. Participants were asked to read the participant information sheet, to sign the consent form, and to fill in a short sociodemographic questionnaire*.* All participants were also given a short guide including the session’s objective, the definitions of “menstrual health” and “menstrual inequity”, the structure and timings of the session, data protection information and the research contact details of the research team. This information guided and supported participants at the start of the session (e.g., to become aware of potential definitions for menstrual health and inequity). Then, the researchers explained the dynamics of the session to all participants and asked for a volunteer in each group to act as a “host”. This role consisted of taking notes and mapping out the discussions during the World Café session and making a short presentation in the plenary session at the end of the World Café.

The second part of the session consisted of three rounds of discussion. In the first round all three working groups engaged in a constructive discussion based on the following question: *“Based on your experience, what are the priority areas to focus on to address menstrual inequity and promote menstrual health?”.* After a 30-min discussion, all participants except the one acting as host were asked to switch rooms for the second round. Following the World Café method this is done so that participants can collectively contribute to the discussion taking place in each table/room. At the start of the second round, the host briefed the rest of the group on the discussion the previous group had. Participants could briefly add onto the feedback given by the host, before moving into discussing the question corresponding to the second round. This was: *“If you could design and implement a menstrual equity intervention, what would be the objectives?”*. Just as the first round, all participants except the host moved tables/rooms for the third round after 30 min. The same procedure was done as in the second round. The third round consisted of addressing the following question for another 30 min: *“What actions would be necessary to reach the objectives set (in the second round)?”*.

The third and last part of the World Café session was a plenary session where all participants and researchers gathered. Each host gave a five-minutes presentation on the discussion held at her table/room, and listed the actions identified throughout the three rounds and especially in the third round. A final discussion took place before ending the session.

#### Data analysis

Data were analysed based on a Framework Analysis [27, 28] approach. A first stage of the analysis consisted in analysing data per each participant group separately. Then, the analyses were merged to construct an analytical framework including data from both women and PWM, and the group of professionals and activists. Data analysis was led by LMP, AGE, ASH, CJA and CMB, and discussed with all authors.

The following steps were taken in each stage: (1) Familiarising with the data through listening to audio recordings and reviewing research notes; (2) Developing an analytical framework, which included four categories, based on research data available and previous evidence; (3) Applying the analytical framework so that data was assigned to each framework category and subcategory; (4) Charting data into the framework matrix, which involved summarising data for each category and subcategory; and (5) Interpreting the data, which was done through meetings and discussions with all authors [27, 28].

## Results

Through the analysis of both studies, six categories were identified for the analytical framework: 1) Debunking menstrual taboo and stigma through resignifying menstruation and the menstrual cycle; 2) Integrating inclusive and holistic menstrual education in school curricula and mass media platforms; 3) Reframing healthcare services to navigate towards integrative and agentic menstrual consultations; 4) Ensuring the access to menstrual products and menstrual management facilities; 5) Menstruation, productive work and menstrual self-care; and 6) Intersectionality and participatory-based research as the foundation for evidence-based menstrual policies. A summary of policy and community actions based on the analysis of both participant groups is available in Table 3.

**Table 3:** Proposed menstrual policies by areas of action

General menstrual policymaking	Create internal political structures so that menstrual policies can be devised and sustained long-term
	Develop participatory-based menstrual public policies, so that communities can be actively involved in menstrual policymaking
	Men and people who do not menstruate should also be a target of menstrual policies (e.g., to work on menstrual taboo and stigma among men)
Menstrual education, taboo and stigma	Integrate intersectionality-based menstrual education in primary schools, high schools, and universities. It should be first offered during primary school and before menarche Education should focus on debunking menstrual taboo and stigma, on raising body awareness (to be able to identify menstrual health problems) and foster menstrual care, and on all stages of menstrual experiences (i.e., including perimenopause and menopause) Educational resources should be available to all regardless of their socioeconomic context
	Improve and promote menstrual health and equity education among health professionals and other professionals in education, social and policymaking
	Promote menstrual knowledge to the population (and particularly to families and parents) through community workshops with health professionals
	Involve community agents in menstrual education in healthcare centers
	Raise awareness on the needs of specific populations (LGTBIQ + , vulnerable migrants, functional diversity, socioeconomic vulnerable people). Ensure the representation of the diversities in menstrual experiences, including those of gender non-conforming individuals
	Implement mass media and public awareness-raising campaigns (e.g., on women and PWM’s rights, disseminating “menstrual health” and “menstrual inequity” definitions, and informing about menstrual products)
	Create a specific information campaign to inform about the quality and potential harms of menstrual products
	Disseminate menstrual information through social media networks, especially for young people
	Create community spaces for social support on menstrual care and to foster emotional health. For example, community agents may foster menstrual education amongst people who do not (or cannot) access healthcare services
	Consider the creation of public spaces for menstrual education and management during municipal festivities
	Projection of menstrual-themed films and series on public TV channels
Menstrual healthcare	Develop a health strategy which includes menstrual health actions
	Restructure the architecture and design of healthcare centres to transform them into more welcoming and less hostile spaces
	Promote the creation of multidisciplinary professional teams that recognize gender and functional diversity, and that consider diverse populations (especially LGTBIQ + , migrant and socioeconomic vulnerable communities)
	Addressing menstruation and the menstrual cycle as signs of health in health consultations
	Foster agentic menstrual healthcare services (e.g., ensure and enable women and people who menstruate to make their own informed decisions)
	Demedicalise and depathologise the menstrual cycle and menstruation in healthcare services (e.g., go beyond medicines prescriptions and offer integrated health options)
	Ensure equal access to good-quality and agentic menstrual consultations, regardless of socioeconomic status and race
Menstrual products and menstrual management	Apply tax reductions on menstrual products. This should include cloth-made reusable menstrual products^*^
	Dispense free menstrual products in public spaces such as schools, universities, healthcare services, public toilets or community pharmacies Especially ensure the accessibility of products amongst socioeconomically vulnerable populations. Make free products also available at food banks, Social Services and prisons
	Ensure students can access kits with spare clothes in schools
	Adapt toilets to menstrual management needs. Ensure the provision of soap, toilet paper, menstrual products, a sink with running water, a bin to dispose menstrual products, and a hanger for clothes in menstrual management facilities. These should also be clean, have a well-functioning lock to ensure privacy and safety
	Adapt toilets for menstrual management so that are inclusive to gender non-conforming menstruators
	Develop a benefits system for socioeconomically deprived women and people who menstruate, to ensure affordability to menstrual products
	Promote the use of reusable menstrual products
	Develop new and innovative menstrual products considering functional diversity
Menstrual care in workplaces	Work on developing and implementing adequate and non-stigmatising menstrual leave options
	Ensure workplace allow for flexible working hours during menstruation
	Ensure the option of teleworking during menstruation (if applicable to the job characteristics)
	Create and adapt workplace spaces to the menstrual management needs (e.g., adapt toilet facilities and rest areas to promote menstrual self-care)
Menstrual research	Create a menstrual health and equity repository, to share knowledge, resources and research data
	Conduct participatory-based research on menstrual health and (in)equity
	Guide menstrual policymaking though research processes
	Conduct research based on (peri)menopause

*Data collection was conducted before the Spanish Government announced the reduction of taxes applied to menstrual products in Spain. However, reusable cloth-made products (e.g., reusable pads) are still taxed as a clothing item and taxes have not been decreased for this product

### Debunking menstrual taboo and stigma through resignifying menstruation and the menstrual cycle

One of the most prevalent claims women and PWM shared was around tackling menstrual taboo and stigma, followed by mentions of menstrual discrimination. They considered speaking up about menstruation imperative to address prevailing taboo and stigma, as well as addressing menstrual taboo and stigma in the workplace, and being able to decide practicing free bleeding without shame. However, as P_WPWM_1 expressed, participants often navigated between the need for a “menstrual breakthrough” and the ingrained taboo they had internalised themselves:

“Not leaving it (menstruation) as a taboo (…) It is something that happens and that’s it and you do not decide it (…) maybe improving it so that it is further spoken, so that women can express what happens to us […] But of course society is what it is, so… if you are in the street you need to have your tampon, you need to have your pad (…) she’s not gonna be out stained, you’re not gonna go around smelling, like her [referring to the woman in one of the photographs shown in the photoelicitation].”—P_WPWM_1.

Likewise, professionals considered menstruation and the menstrual cycle to be stigmatised and taboo processes, resulting from patriarchal societal structures. They suggested the need to transform how the menstrual cycle is socially understood, to change menstrual symbolism towards a more respectful and “positive” one. They claimed how this should be done based on a feminist and intersectional perspective (i.e., taking into account different axes of oppression and discrimination) and understanding menstruation and the menstrual cycle as indicators of health in women and PWM. Eradicating menstrual taboos and stigma was also considered imperative. However, one participant offered a reflection on how taboos could have been (and are) protective mechanisms against certain gender role expectations (e.g., women having to be fully responsible for reproductive work):

“The taboo prohibits things, but the taboo also protects you from things. If we look at it from distance, it was also a way for you to get away from hard work of women for a few days. So, even in cultures where there is still no time to take your freedom to rest, taboo is sometimes the only thing that allows you to… ‘I can't cook, I can't…’”—P_PA_22.

Following the above statement, a participant pointed out that taboos were present in all cultures but in different degrees of expression, questioning the nature of “cultural differences”:

“I think that sometimes we see cultural diversity as something that makes us very different. Here it is mayonnaise and in another place it is 'do not touch the plants [referring to myths around menstruation]. This makes us very equal. The patriarchal society is the same in different degrees of expression. So, a strategy to break the cultural difference would be to make us feel that we are on the same plane”—P_PA_3.

Ideas that women and PWM shared on how to lessen menstrual taboo and stigma included engaging with social media influencers to normalie menstruation. In line with this, P_WPWM_24 claimed the need to make menstruation, and the social burden that comes with it, visible in a non-stereotypical way to attain menstrual health. He (P_WPWM_24 preferred masculine pronouns) explained how menstruation is stereotypically portraited in the media as either discomfort-free or a “cyclical horror”, failing to make visible the wide array of menstrual experiences. His experience was also shared by P_WPWM_11, who thought the media had a responsibility in not only portraying what could be considered “*an idyllic menstruation”.* P_WPWM_24 particularly mentioned the need for all menstrual bodies, in all their diversity (referring to gender non-conforming menstruators) to also be publicly visible, for instance in the media:

“There are a lot of generalisations, speaking about women or speaking about a certain type of women that menstruate in a certain way (…) There are many realities and… I feel that it is important that this gets put on the table”—P_WPWM_24.

In line with this, professionals considered that menstrual policies should focus on the needs of each population (e.g., people with functional diversity, socioeconomic vulnerable people, migrant populations, and LGTBIQ + communities). Regarding the stability of these policies over time, one participant claimed the creation of internal political structures that ensure the maintenance of menstrual policies:

“To guarantee that the institution maintains this and not depending on which deputies (…) That there is a profile that is mixed between jurists and that has a gender perspective and that can make impact reports. (…) At least, you have the office and if the legislature changes you have it there within the administration”—P_PA_1.

Besides, as women and PWM exposed, creating spaces to express and make their menstrual experiences visible could lead to getting social support. P_WPWM_18 and P_WPWM_30 narrated how addressing menstrual taboo and stigma could have a significantly positive impact on the lived menstrual experiences and emotional health among women and PWM. Further, P_WPWM_18 expressed that debunking menstrual taboos could lead to:

“Taxes (on menstrual products) to be reduced or that we could get products for free (…) and also discrimination for having our period, or some insecurities, self-esteem problems because of having a period and things like this, that I really believe that happen quite often. And in the end, from my opinion, they come from (menstruation) not being perceived as something natural and the fact that it is something hidden”—P_WPWM_18.

Moreover, P_WPWM_29 perceived menstrual taboo to be more deeply rooted among men, claiming that policy strategies should also target men. Involving men and people who do not menstruate (PWNM) in promoting menstrual health and equity was imperative for some to encourage men and PWNM to debunk menstrual taboos, promote empathy, and give social, emotional, and instrumental support to women and PWM:

“So male adolescents should be capable, so that if they see a girl that has stained her pants, tell her, hey do you need anything? Do you want me to go look for something? Instead of “wow!”—P_WPWM_7.

### Integrating inclusive and holistic menstrual education in school curricula and mass media platforms

The need for timely, adequate, and holistic menstrual education was, overwhelmingly, the most predominant claim made by women and PWM. P_WPWM_17 pointed out that menstrual education should not be relegated to the family context, but to be offered at schools, to ensure and standardize the access and quality of menstrual education. They perceived menstrual education as the first step towards achieving menstrual equity and promoting menstrual health. According to professionals, menstrual education should be integrated into educational spaces (e.g., primary schools, high schools, and universities). They emphasised that menstrual education should go beyond one-time workshops and become an integral part of school curricula:

"If we focus on education, there must be a plan that is not an hour and a half talk because sometimes you open the Pandora’s box of 'How did you experience your first menstruation?' (…) It must be an (educational) program at least two or three times (a year)."—P_PA_9.

Some professionals referred to the implementation of a health subject in school curricula before menarche, while others suggested addressing menstruation and the menstrual cycle in a cross-cutting manner within existing subjects in school programs. Professionals agreed on the need for a pedagogical intersectionality perspective on menstrual education. While some women and PWM thought of menstrual education within the framework of sexual and reproductive health (e.g., to prevent unwanted pregnancies), others called for critical and feminist-based menstrual education. Based on their narratives, menstrual education should focus on debunking menstrual taboo and stigma. Learning how to manage menstruation, menstrual pain, and understanding the wide range of menstrual products available were other topics that women and PWM believed should be included in menstrual education. P_WPWM_18 also mentioned that menstrual education should encompass menopause. Besides, they believed that menstrual education should focus on promoting body awareness to identify potential menstrual health problems and include the social sphere of what it means to menstruate:

“And I thought, why don’t they do this at school? (…) now when I read certain things, like the fact that periods aren’t painful, I think ‘what do you mean periods aren’t painful?’ And I think ‘why wasn’t I told this in school?’ Periods aren’t painful. (…) Why did I find out when I ‘m 30 years old?”—P_WPWM_6.

Menstrual information was perceived by P_WPWM_27 to be increasingly more available in the last few years, given that there are more books published around menstruation and sexual health, and “red tents” and menstrual workshops are more widely available. However, she also pointed out how these resources were only accessible to those who are motivated and have the financial means. In line with this, professionals mentioned that educational strategies should be inclusive of different menstruating realities (e.g., people with functional diversity, socioeconomic vulnerable people, migrant populations, and LGTBIQ + communities) and that they should incorporate an intercultural approach that acknowledges cultural diversity:

“We did a talk to families at school about this (menstruation and menstrual cycle). Of course, very few (family members) came, the language was a barrier, because of course, we have a lot of immigration there. There were mothers who came with good intentions, but they didn’t understand the message well (…) (It would be) Better if we made it a bit multilingual”—P_PA_6.

Both women and PWM and professionals agreed on that menstrual education should be designed and implemented regardless of sex/gender, so boys and PWNM should also be targeted for menstrual education actions. Besides, a few women who were mothers shared their concerns about how to educate their children, regardless of their sex but particularly referring to girls, *“How will I address it when my girl has it* (menstruation)*?”*—P_WPWM_33. One participant also mentioned that parents’ concern about teenage pregnancy is the reason why, as a mother, the first thing that will come to her mind to explain to her daughter is that menarche means she can get pregnant* “(…) then we will then see if you have polycystic ovaries or you don’t have your period, or you get acne (…)”*—P_WPWM_33*.* Further, P_WPWM_34 deduced that menstrual education should also be directed to parents: *“Sometimes I think that we should have a course for parents, on how to educate our children or something like that […] To educate. Having children is easy but educating is very difficult”*—P_WPWM_34.

Another demand from professionals was to launch a communication campaign through the mass media and social networks, especially addressed to young people. They explained that these campaigns should have an emphasis on women and PWM rights, disseminate the definitions of “menstrual health” and “menstrual inequity” and inform about menstrual products. Further, P_PA_19 mentioned that a critical stance on the potential harm of non-reusable menstrual products should also be disseminated to the wider population:

“Tampax tampons and all these brands that say: cotton, polyester, cotton that is not cotton… How is it possible that politics is not there…? If they (products) are harmful products to our health, why isn’t there greater visibility of what we’re putting in?”—P_PA_19.

### Reframing healthcare services to navigate towards integrative and agentic menstrual consultations

Another central tenet for women and PWM was the need to improve the access and quality of healthcare services for menstrual health. According to them, health services should be designed and implemented with the menstrual cycle and menstruation as general health indicators and tools for health promotion in mind. Moreover, women and PWM thought that menstrual health should be addressed in healthcare services in a more holistic (and less biomedical) manner. For instance, using hormonal contraceptives was perceived by P_WPWM_6 as a barrier to menstrual awareness and body literacy, supporting the need for menstrual health consultations to go beyond medical prescriptions. Demedicalising and depathologisating menstruation (pregnancy and the climacteric) was another of their demands. As P_WPWM_27 expressed, medicalisation created a sense of disempowerment for her:

“It (medicalisation) creates some sort of disempowerment (…) that perception that makes you feel like something bad is going on and that, also, you have to control it with a pill or something medical. And, why? If it is a natural process for women, right?”—P_WPWM_27.

Based on the accounts of professionals, healthcare centres are often perceived as hostile spaces, especially for young people and vulnerable populations. Thus, they suggested architectural and design changes to make healthcare spaces more welcoming and facilitate menstrual consultations. They believed that if health centres were seen as health promotion structures and not just as spaces accessible only during illness, they would be more accessible to potential users for menstrual consultations. One participant also commented on how gynaecological services were often were not available for women who were no longer menstruating:

"My mother-in-law, 85 (years), tells me 'oh, my vulva itches, it is dry, but the gynaecologist told me not to return at 65 (…) you are not a woman because you menstruate, but until you die’”—P_PA_9.

Besides, women and PWM in our study considered it essential to improve professionals’ knowledge and attitudes towards service users and menstrual-related consultations. Some participants also referred to the generalized neglect of menstrual health needs by healthcare professionals, and not feeling listened to:

*“I believe that the first thing it to listen to women, right? Whatever happens to her, whatever she explains to you on menstruation, don’t ignore it and if she tells you that it* (menstruation) *hurts her, well then let’s explore it, right?”*—P_WPWM_12.

P_WPWM_27 was also critical towards health professionals who made it challenging for women and PWM to take agentic informed decisions:

“With doctors there’s also a lack of clarity. For example, with hormonal contraception they don’t explain to you that, you do not actually have a cycle and that it is a bleeding that is caused by a pill, but you are not going through the same process afterward. All these things are not explained to you clearly, and of course you don’t have the resources to know if you need it (hormonal contraception) or not”—P_WPWM_27.

To increase awareness of menstruation throughout society, professionals mentioned that university curricula should also incorporate menstrual health and equity into educational, health, social and political studies. In this regard, some participants considered relevant for healthcare professionals to strengthen menstrual health community-based actions, as they found that workload pressures limited their capacity for community health actions. At the same time, offering menstrual health talks by midwives was seen as an appropriate measure to inform to communities. One participant explained that the role of community agents, understood as people who serve as a "bridge" between the healthcare services and the community, could be a powerful resource to disseminate menstrual information to people who do not (or cannot) access primary healthcare centres for various reasons (e.g., language barriers):

“I think that as midwives, it is a high priority to go out, to be more in the street, in neighbourhood associations (…) to be in contact with all the agents in the neighbourhood, because we can get into spaces that perhaps we wouldn't get into if we didn't leave the primary healthcare centre and perhaps reach a population that doesn't come to the primary healthcare centre”—P_PA_11.

Something else that emerged in the narratives of women and PWM was that access to healthcare should be ensured for those living in socioeconomically deprived situations. Although she did not see it as problematic, P_WPWM_2 referred to the unequal access to healthcare opportunities:

“Equal conditions for everyone (…) My opinion is that if you (…) (have) money, (you can) pay for it (healthcare) and that’s all […] The person who can’t (afford private healthcare) goes to social security [public healthcare] and that’s it”—P_WPWM_2.

Besides, P_WPWM_31, who worked as a cultural mediator in healthcare services, stressed the need to respect women’s decisions regardless of their sociocultural background. She explained a situation she had experiences where a gynaecologist denied a Pakistani woman a consultation when she refused to be seen by a male trainee medical student: *“And she got angry and left the consultation there, and she said that she had to leave and get a consultation with someone else”*—P_WPWM_31*.* The participant explained that the doctor had wrongly assumed that it was the patient’s husband who refused the male student to be present in the consultation, and that the patients’ husband filed a formal complaint. P_WPWM_31 call for respecting women’s decisions and inferred the need to address cultural and racial discrimination in healthcare services:

“We must respect the person’s opinion. We cannot complain because she does not want to be seen by a man (…) there are also male doctors in Pakistan who do surgeries and all that, and there are women who allow it and other women who do not […] The doctor started to ask, and why don’t the husbands allow it? (…) but if she is here in Spain, she should not allow this. I know that she is in Spain, but it the one who does not want to”—P_WPWM_31.

Professionals also pointed out the frequent disregard/neglect in healthcare services of the LGTBIQ + community, young people, migrant populations, and socioeconomically vulnerable individuals. For this reason, they suggested creating multidisciplinary teams to guarantee an adequate consultation approach that takes diversity into account:

“So it is very different if there is (in healthcare centres) a person from that community, if there is a deaf person, if there is a person from that culture… People with cognitive diversity.”—P_PA_21.

### Ensuring the access to menstrual products and menstrual management facilities

Women and PWM made consistent claims on the need to improve access to menstrual products, given that they were considered essential goods: *“that everyone can access, right? (…) a basic product that all women could access”*—P_WPWM_29. A few participants also expressed their views on promoting the use to reusable menstrual products, such as the menstrual cup, for both the environment and health-related reasons: *“they* (single use products) *have got a lot of chemicals that are hormonal disruptors”*—P_WPWM_21. According to their narratives, access could be enhanced by providing free menstrual products (e.g., in public toilets), reducing their cost (in particular by lowering taxes) or making state benefits available to purchase menstrual products available for socioeconomically vulnerable people. P_WPWM_18 shared her views on the link between current taxation of menstrual products and social discrimination based on menstruating, as these products were not taxed as essential goods at the time of the interviews. P_WPWM_21 further discussed feminism and the relationship between political ideologies and people’s attitudes towards subsidizing menstrual products:

“How many women are affiliated to (…) (political party), which is far-right and misogynist (…) because saying this (disagreeing with making menstrual products available for free) means that you disagree with having sexual rights (…) It means being very disconnected from your gender and your sexual identity […] There are many different feminist movements. To me, feminism should simply be that everyone could do whatever the hell they want (…) the important thing is that you are free to choose (…) As long as you are well-informed”—P_WPWM_21.

Professionals also commented on the importance of taking action around the price of menstrual products to address menstrual poverty, for example, by reducing and/or eliminating taxes on menstrual products. One participant pointed out that a 21% tax was still applied to cloth-made reusable menstrual products, despite the fact that other menstrual products are taxed at a reduced rate. Besides, participants discussed the importance of guaranteeing equal access to all menstrual products, avoiding the imposition of some options over others (e.g., not considering the menstrual cup as the best option). They also mentioned the placement of product dispensers in strategic locations, such as social services, primary care centres, pharmacies, prisons, or food banks, to ensure accessibility to vulnerable and hard-to-reach populations. Above all, they considered that cultural, religious, economic, bodily, and functional diversity should be considered to respect individual choices about the use of menstrual product:

“Everything (menstrual products) should be on the table. (…). We should not go with such an exhaustive mantra about what to do and what not to do, that is, am I not feminist enough because now I don't do this (referring to menstrual cup use). All options must be shown”—P_PA_9.

Other women and PWM focused on the availability of menstrual management spaces in public settings. They thought adequate menstrual management spaces should also include a washbasin, a waste bin, and a clothes hanger. These toilets should ensure privacy and safety, have a lock, and be clean:

“Well, facilities in public spaces (…) Here in Spain, for example, there are not many public toilets as in other countries. And why not the possibility of a public toilet for women. Or, just as they have divided men and women, the same for people who menstruate. And an equipped toilet, right? In case you’re using a menstrual cup, to be able to clean it or have accessible products for you”—P_WPWM_14.

Professionals also identified barriers to accessing adequate menstrual management facilities. As a way to minimize these barriers, they suggested the need to adapt public spaces for menstrual management in educational settings (particularly in schools, high schools, and universities), healthcare centres, and workplaces. As women and PWM stated, these facilities should all have access to water and soap, toilet paper, a bin to dispose menstrual products, and even menstrual products available. In school settings, some participants shared the idea of offering spare clothes to students, in case they experienced menstrual blood staining at school. When discussing gender neutral *versus* sex segregated bathrooms, not all participants shared the same viewpoint. Some participants felt that toilets should be gender neutral to be inclusive of non-conforming menstruators and especially to support people who may experience "gender dysphoria" or other difficult emotional experiences when having to use sex-segregated toilets. Others believed that sex-segregated bathrooms are necessary to ensure the physical and emotional safety of women. However, participants stated that all bathrooms, whether or not they are sex-segregated, should be adapted to menstrual needs:

“Personally, I think that there should be sex segregated bathrooms, for safety reasons. But all bathrooms should be adapted in case there is a menstruating person to change and clean them well.”—P_PA_11.

### Menstruation, productive work, and menstrual self-care

Women and PWM referred to the need for menstrual policies in the workplace, including menstrual leave, having “rest days” available, and ensuring flexible working hours. They explained that these policies should be accompanied by processes to normalize and socially accept the need for rest and adaptation of workplaces to the menstrual needs of women and PWM's, so as not to increase menstrual stigma and discrimination:

“To be able to request a day off if you’re not feeling well and not feel ashamed to say it in fron of anyone, right?”—P_WPWM_12.

“To adapt society a little so that menstruation is a reality. Hello, it’s happening, and we need so much… support to… live with it in an easier way”—P_WPWM_13.

Professionals also highlighted the need to adapt workplaces to be respectful of menstrual experiences and needs. They referred to policies such as teleworking or menstrual leave, to support menstrual pain management, although the diversity in working conditions should be considered when developing menstrual measurements in the workplace (e.g., not all jobs offer the possibility of teleworking easily taking bathroom breaks during the working hours):

"In the workplace, bathrooms that are in a good condition (…) this should be part of the idea of optimal working conditions for any job, because in many jobs you can’t even go to the bathroom"—P_PA_20.

Some professionals also questioned the current social and economic model, based on high productivity and efficiency. Within this system, they discussed how menstrual needs could not be met. For instance, work productivity expectations may not be met when experiencing menstrual pain, or they may be met but at the expense of one’s health. For this reason, some participants believed it was necessary to implement work measures that take menstrual experiences into account and challenge the current hegemonic model of productivity:

“Being able to adapt our work dedication, efficiency based on what we can and cannot do. And if you are not as productive as the system wants, then it’s fine. What it [the system] wants is to be ultra-productive”—P_PA_3.

### Intersectionality and participatory-based research as the foundation for evidence-based menstrual policies

Alongside other actions, several women and PWM and professionals called for the need to generate evidence around menstruation and the menstrual cycle. Professionals emphasized the importance of active collaboration between academia, professionals (e.g., from health settings), and the community, promoting participatory action research. In fact, one woman called for menstrual strategies to be designed based on listening to the needs and demands of women and PWM:

“And they should also listen more to people who menstruate when making decisions regarding people who menstruate, this is a logical thing”—P_WPWM_9.

In parallel, another participant explained how: *“it is always about the government, private companies caring for their employees, and then there’s also the role of civil society, social entities, organisations, *etc*. to promote equity, let’s say, between those who menstruate and people who don’t menstruate”*—P_WPWM_8.

Besides, one professional shared her views on how academics rarely focus on translating their findings into community actions and policies that could be beneficial to the population:

“It is a world (the academic) that seeks knowledge that does not interest people too much. It’s always the same. Just like in the political world. Many things are done, many actions, but very few reach (people)”—P_PA_12.

When discussing menstrual research, professionals suggested creating a repository containing research evidence on menstrual health and equity. In addition, this repository could include resources for menstrual education and other useful publications and materials developed by subject matter experts. As inferred from their narratives, research should incorporate a gender-based and intersectional approaches. Inclusion of people with functional diversity and gender non-conforming populations should also be central to menstrual research. Moreover, professionals called for the promotion of research on perimenopause and menopause.

## Discussion

This research provides an overview of the needs of women and PWM, professionals and activists, for menstrual policies and community-based actions to address menstrual inequities and promote menstrual health in Catalonia, Spain. Policymaking needs were identified to tackle menstrual taboo and stigma, to enact menstrual education strategies, to improve access and quality of menstrual healthcare services, together with the access and affordability of menstrual products and menstrual management facilities. Participants also referred to workplace policies, such as the menstrual leave, and the need to critically question current socioeconomic systems to ensure menstrual health and equity. Based on participants’ narratives, intersectionality should be central to menstrual policymaking, and the needs of vulnerable populations should be particularly considered. To achieve these aims, it is imperative to improve participatory strategies to menstrual research and policymaking.

Overall, findings were quite similar between participant groups, which may be a sign of menstruation being more present in social and political discourses and imageries. Another reason may be due to the division between woman/PWM-professional/activist is rather a virtual one, given that all participants who “act” as professionals or activists were also women and had menstrual embodied experiences. While we may initially fall into trying to “divide and compare” participants’ narratives, it was fruitful to unite and enrich them. Main findings are discussed in this section. We will refer to “participants” when referring to both participant groups and distinguish between groups where necessary.

In our research, participants particularly emphasized the need to implement menstrual policies to tackle taboo and stigma, by reshaping detrimental societal narratives [29, 30]. These measures should encompass the recognition of menstrual stigma as originated from gender and menstrual injustices and working towards dismantling it shifting gendered power dynamics [17, 31, 32]. As Alhelou et al. [9] suggested, menstrual policymaking should address the complex roots that ground menstrual inequities (e.g., menstrual stigma and gender inequities), rather than focusing on quick and tangible solutions. However, a notable lack of clarity persists regarding on how policymakers should effectively address menstrual stigmatization [33]. Although participants in our study suggested some ideas on how to address menstrual stigmatization, these focused on “raising awareness” and “promoting education”. Instead, and as already suggested in previous evidence, context-specific actions and policies targeting gender and menstrual injustices is most required. In other contexts, such as India, Kenya, Senegal, and USA, several policies aimed to dismantle menstrual stigma, but they only helped as a first step in “breaking the silence” surrounding menstruation [9, 34]. Hence, it is crucial to comprehend how menstrual stigma and discrimination operate, to establish suitable frameworks for the design, implementation, and evaluation of effective measures.

Participants highlighted the importance of integrating menstrual education into formal education curricula. This education should be promoted through an intersectional lens, considering the diversity in menstrual experiences and needs and adopting holistic perspectives [31]. As previous research has highlights, this means to move beyond purely biomedical approaches and prioritizing body literacy [35] and agency. In line with participants' narratives, available evidence and previous menstrual education policies have indicated that it is crucial to ensure educational menstrual interventions regardless of sex/gender, so that they are inclusive of boys and PWNM [36] and reinforce education about perimenopause and menopause. While some educational interventions conducted with adolescents in different contexts have proven effectiveness in increasing knowledge about menstruation and the menstrual cycle [37–40], their impact on school participation and experiences of menstrual stigma and discrimination remains uncertain [41, 42]. Moreover, it is essential to broaden research beyond adolescence and focus on pre-menarche and adult populations, to promote accurate and comprehensive menstrual education across different life stages. As some mothers in our research highlighted, there is the need to equip adults and families with enough resources to provide menstrual education to younger generations. This is particularly relevant as most women and PWM first learn about menstruation from family members [6, 14].

Participants in our study also mentioned the need for communication campaigns through mass media and social media networks, which may support menstrual education especially amongst young people but also in adulthood [6]. However, mass and social media can be a double-edged tool, because they are powerful in raising awareness and driving political actions, but can also pigeonhole menstruation as menstrual management and perpetuate menstrual stigma [34]. Also, potential drawbacks stemming from the digital divide should be recognized, as it has the potential to exacerbate existing social inequities [43].

In our study, participants also proposed measures focused on ensuring that healthcare professionals adopt a respectful and inclusive approach towards diverse populations and realities, and challenge *menstrual normativity* [31]. To achieve this, menstrual education should also be promoted and strengthened amongst healthcare professionals, particularly in primary healthcare and specialised services, and multidisciplinary teams could be established. Consultations should be free from judgmental and discriminatory practices and foster agentic informed decisions by women and PWM [6, 14]. As women and PWM in our research emphasized, healthcare services should turn towards integrative health and question the prevailing medicalization and pathologisation of healthy processes such as the menstrual cycle [17, 32, 44]. While menstrual education policymaking has been emphasized, menstrual health and equity healthcare policies are significantly scarce.

Another concern participants shared was related to the access to healthcare services (and education) amongst vulnerabilised populations. In line with this, discussing the *cultural racism* that often arises in menstrual debates and policymaking is particularly relevant. This form of racism refers to the prejudices and discrimination enacted towards individuals based on “their culture” and “cultural differences” [45] (e.g., considering that menstrual taboos are only present in other cultures), rather than recognizing other axes that cause these differences, such as poverty. One participant from the group of professionals in our study mentioned that menstrual taboos are present in all cultures. This challenged *cultural racism* discourses that were apparent amongst other participants, who discussed menstrual taboo and stigma as relative or more prevalent in “other cultures”. Similarly, one interviewed woman discussed racist and discriminatory practices in healthcare services. Discourses of *cultural racism* are also present in the literature and have positioned women and PWM as “victims” of “an oppressive” culture, failing to understand the diverse menstrual meanings and practices. Based on Winkler and Bobel’s work, this is often framed in comparison to “white/Western culture”, which is often portrayed as normative, desirable, and progressive, while the global South is presented as backward, “savage”, and regressive [46]. Questioning and debunking longstanding structural privileges and oppressions, open dialogues and cultural sensitivity are unquestionably essential to ensure agentic and effective interventions and services [47].

Besides, participants generally advocated for addressing menstrual poverty [10], and referred to policy actions to reduce taxes and general price of menstrual products to improve their affordability. However, a study evaluating tax reforms for menstrual products in the European Union member states found that, while tax reduction alleviates the financial burden on women and PWM, it may not be the most effective solution to improve access to menstrual products [48]. While reducing or eliminating menstrual taxes on menstrual products can contribute to addressing menstrual poverty, it is important to highlight that it cannot be effective as a stand-alone policy. Therefore, it must be accompanied by other policies that address underlying social inequities that cause and maintain poverty. Other measures mentioned were placing a system of state benefits for socioeconomically vulnerable populations and dispensing free menstrual products in key public places and targeted to vulnerabilised populations (e.g., women in prisons). As for menstrual taxes, these should not be implemented in isolation. In addition, although it did not stand out in participants’ narratives, some participants mentioned the relevance of fostering the use of reusable products, not only as a measure for menstrual poverty but to protect human and planetary health [49, 50]. In fact, the Catalan government has recently announced that reusable menstrual products (menstrual cup, reusable pads and menstrual underwear) will be available for free in community pharmacies from the first quarter of 2024. However, this service will only be available to those who have access to a public health card. This means that women and PWM in irregular administrative situation, one of the population groups most at risk of menstrual poverty in Spain [6], will not have access to this service.

Having access to adequate facilities to manage menstruation in public spaces was another main claim participants made. Consistent with previous literature, participants generally agreed that public spaces, including schools and workplaces, should be equipped to guarantee menstrual management and prevent negative health outcomes resulting from unhealthy menstrual management practices [51]. While participants agreed on most attributes of menstrual management facilities, the group of professionals and activists discussed the suitability of gender-segregated versus gender-neutral bathrooms. On one hand, some participants argued for the maintenance of segregated bathrooms to uphold and respect the experiences and needs of women (particularly referring to the need of safety and intimacy). On the other hand, some participants advocated for gender-neutral bathrooms to be inclusive of gender non-conforming menstruators. Studies have shown that transgender [52, 53] and non-binary [53] individuals face barriers when accessing public bathrooms, which are amplified when they are menstruating [14, 53]. As research shows, this could worsen feelings of gender dysphoria and negatively impact emotional health. While the debate may still be open, actions must be taken to ensure the rights of all people who menstruate to healthy menstrual management and care.

The menstrual leave, and other workplace menstrual policies, often emerged in our participants’ narratives. However, recent research [14, 54] has identified barriers that could turn menstrual leaves, not only into ineffective but damaging to the health and wellbeing of women and PWM. For instance, the menstrual leave may be perceived as “unfair” and detrimental to business productivity. For this reason, these menstrual policies should be considered carefully as they may increase menstrual stigma and gender discrimination. For instance, it could reinforce social imageries of women being “weaker” and “more expensive” for employers [55], and sustain gender pay gaps and “the glass ceiling” [54, 56]. Nonetheless, the solution may not lie in refraining from adopting menstrual measures in the workplace. Instead, and as one participant rightly articulated, policies and community actions should take a critical stance on socioeconomic models that do not leave space for menstrual care and contribute to menstrual inequities [6, 57, 58].

A central tenet to this research encompassed incorporating an intersectional lens [3] to menstrual policies. Intersectionality is particularly interested in exploring how these systems of privilege and oppression occur in patriarchal, colonial, and capitalist systems. Therefore, an intersectionality approach would require focusing on how societal privileges and oppressions intertwine and shape individual and collective experiences. Participants acknowledged that policymakers should account for the needs of populations that have been traditionally marginalised, especially based on race, social class, migration status, sexual orientation, and gender identification. This is supported by previous research in our context, as menstrual inequity is particularly experienced by vulnerablilised populations, such as socioeconomically deprived women and PWM, vulnerable migrants and gender nonconforming individuals [6]. However, the inclusion of vulnerabilised populations is not enough to disarticulate menstrual inequities. Instead, policies should recognize and address how power structures generate and maintain systems of privileges-oppressions. Thus, intersectionality-based policies need to be designed and implemented carefully to avoid the risk of instrumentalization, abuse, and misuse of intersectional approaches [59].

As importantly, participatory methodologies [60] need to be incorporated in menstrual research and policymaking, as they can bring the improvement of quality, empowerment, capacity building and sustainability in research processes [61]. It is essential that research and policies are guided by processes that respond to women and PWM needs, rather than being driven solely by the experiences and “expertise” of academics and policymakers. However, power relationships in research and policymaking often persist, creating barriers to ensure participation of excluded and marginalised menstruators in decision-making processes. Efforts must be made to address power imbalances, promote equitable participation, and create spaces for engagement of diverse communities into the research and policymaking. Likewise, menstrual strategies should not only be fostered within institutions but also within communities. Moreover, it is essential to strengthen evaluations to assess the effectiveness of menstrual policymaking. Improving methodologies for evaluating menstrual health interventions and allocating sufficient funding for evaluation are crucial steps to ensure evidence-based and effective policies and programs before wide-scale dissemination [62].

### Strengths and limitations

The main strength of this study is that it provides innovative qualitative data on the needs for menstrual policymaking and community-based actions in Catalonia. The findings of this study could raise awareness and direct researchers and policymakers. Another strength is the use of the choice and merge of methodologies, including photoelicitation techniques and the World Café approach While action-based participatory research is needed, this study was a step in understanding how to unite menstrual needs amongst women and PWM and professionals and activists with expertise in menstrual health and equity.

Some limitations need to be acknowledged. While participants’ discourses expressed a wide range of political actions, detailed explanations on each policy (e.g., the methodology of implementation) were not provided. Consequently, this study could not deepen the debate on certain policy issues. Another potential limitation of our study is the lack of diversity among participants, particularly regarding socioeconomic status, race, functional diversity, gender identity, and other characteristics. This should be acknowledged, and findings need to be adequately contextualised.

## Conclusions

This research provides an overview of the needs of women and PWM, and professionals and activists, regarding menstrual policies and community-based actions in Catalonia (Spain). Our study highlighted the need to include actions to address menstrual taboo and stigma, promote menstrual education that goes beyond the hegemonic biomedical prism, improve access and quality of menstrual health services, alongside policies to ensure adequate menstrual management facilities in public spaces and access to menstrual products. Policymaking should also focus on how to ensure menstrual management and care in workplaces. Menstrual policies and community-based actions should be framed within intersectionality, to consider how power and oppressive societal structures influence menstrual experiences. Designing and implementing multifaceted policy and community-based actions to address menstrual inequities and promote menstrual health is necessary. This research offers some guidance to researchers and policymakers on how to lead and design evidence-based menstrual policies. Beyond the contents of such policies, policymakers need to commit to adequately evaluating policy outcomes and avoid tokenism, as well as reflect on potential drawbacks of policy strategies.

### Supplementary Information

**Additional file 1: Material S1.** Critical Appraisal Skills Programme (CASP) criteria. **Material S2. **Interview topic guide. **Material S3.** Photographs used for the photoelicitation interviews. **Figure S1.** Marathon runners. Kiran Gandhi in the London Marathon with her friends. **Figure S2.** Mexican congresswomen and Menstruación Digna México (@dignamx) representatives.

## Data Availability

The datasets generated and analysed during the current study are available from the corresponding author on reasonable request. Datasets are not publicly available to maintain participants’ anonymity and confidentiality.

## Abbreviations

PWM: People who menstruate
PWNM: People who do not menstruate
P_WPWM_: Participant “women and people who menstruate”
P_PA_: Participant “professionals and activists"
