# Supplementary material for: Perspectives on menstrual policymaking and community-based actions in Catalonia (Spain): a qualitative study
Source: Reprod Health. 2024 Jan 4;21:1. doi: 10.1186/s12978-023-01730-9 (PMC10768382; doi:10.1186/s12978-023-01730-9)
Supplement: Supplementary file 1 — Additional file 1: Material S1. Critical Appraisal Skills Programme (CASP) criteria. Material S2. Interview topic guide. Material S3. Photographs used for the photoelicitation interviews. Figure S1. Marathon runners. Kiran Gandhi in the London Marathon with her friends. Figure S2. Mexican congresswomen and Menstruación Digna México (@dignamx) representatives. [file 12978_2023_1730_MOESM1_ESM.docx]

Additional file 1

Material S1. Critical Appraisal Skills Programme (CASP) criteria

1. Was there a clear statement of the aims of the research?
   1. Yes. The aim of the study is available at the end of the Background section (page 5).
2. Is a qualitative methodology appropriate?
   1. Yes. The need and relevance of using qualitative methodologies has been described throughout the article, and particularly in the Background and Methods section.
3. Was the research design appropriate to address the aims of the research?
   1. Yes. The design and theoretical approaches taken to conduct the research have been appropriate and clearly identified in the Methods section (pages 5-6).
4. Was the recruitment strategy appropriate to the aims of the research?
   1. Yes, the recruitment strategies have been outlined in the Methods section (pages 6-7).
5. Was the data collected in a way that addressed the research issue?
   1. Yes, data collection strategies have been defined and justified in the Methods section (pages 6-8).
6. Has the relationship between researcher and participants been adequately considered?
   1. Yes, authors applied reflexivity throughout the research, so they have continuously reflected on how their own experiences, perceptions and privileges have affected the relationships with participants. This has been outlined in the Methods section.
7. Have ethical issues been taken into consideration?
   1. Yes. Ethical approval was obtained from IDIAPJGol Ethical Committee on 21st Nov 2020, Ref 19/178-P.  Written and verbal consent were obtained prior to participation and audio recordings All participants were made aware of their participation being anonymous, confidential, and voluntary, along with their right to withdraw consent to participate at any given moment until data analysis. Ethical approvals have been included in the Declarations section of the manuscript.
8. Was the data analysis sufficiently rigorous?
   1. Yes. Data analysis has been triangulated, formulated, discussed with the research team, and discussed again and reformulated before reaching final data analysis. The analysis process has been described in detail to ensure transparency. Data analysis is available in the Methods section (page 8).
9. Is there a clear statement of findings?
   1. Yes, results are clearly stated and discussed. The conclusion along with the research, policy, and practice recommendations clearly explain the findings.
10. How valuable is the research?
    1. The main strength of this study is that it provides, to our knowledge, the first qualitative data on the identification of policies and community-based actions to address menstrual inequity and promote menstrual health in Catalonia. The findings from this study could raise awareness among researchers and policymakers in continuing thinking about menstrual policies and community-based actions that should be implemented in our context.

Material S2. Interview topic guide

1. How would you describe yourself?
2. What is menstruation for you?
   1. How do you feel about it?
   2. What do you feel in relation to menstruation?
   3. What are the negative aspects of it for you? And the positives?
3. What is menstrual health for you?
4. How was it the first time that you menstruated?
   1. How did you feel?
   2. How were the reactions of people around you?
   3. Who did you speak with?
   4. Have you felt that you could speak to your family about menstruation?
   5. Did you feel ready to start menstruating?
5. Usually, how is your menstruation like?
6. Do you notice any physical and/or emotional changes throughout your menstrual cycle (so between one menstruation and the next)?
   1. If so, what changes do you experience?
   2. How do you feel about these changes?
7. How have you learnt about menstruation?
   1. Do you feel that you have enough information?
   2. How have you informed yourself since you started menstruating until now?
8. [Show photography 1, marathon runners]
   1. Could you describe this photograph?
   2. What do you feel looking at it?
9. Do you think that menstruation is a taboo topic (a topic that people do not talk about or is ashamed of doing it)?
   1. If so, why do you think that is?
   2. Does that happen to you?
10. How do you think menstruating may impact your daily life?
11. What activities do you avoid when menstruating (e.g., because of menstrual pain or being ashamed)?
12. Some people believe that menstruating may be a disadvantage (in relation to men/people who do not menstruate) at school or at work, what do you think about this?
13. Has it ever happened to you that you stop doing your day-to-day activities (e.g., not going to work or school) when menstruating? Can you tell us about it?
14. Some participants tell us that they feel the need to rest when they are menstruating and they are not as productive or have trouble concentrating, what do you think about this?
15. Have you ever sought help from a healthcare professional for any menstrual-related questions or issues?
    1. How has your experience been?
    2. Have you spoken to someone else about it?
    3. Have you attended any other services/sought help from someone else?
16. What do you think about the price of menstrual products?
17. Some people have told us about having economic problems and not being able to afford menstrual products, has this ever happened to you?
18. Has it ever happened to you that you could not choose what menstrual products to use due to financial issues?
19. Has it ever happened to you that you could not afford other goods or services to be able to get menstrual products?
    1. If so, how have you felt about it?
20. How have you access menstrual products generally?
    1. Have you ever had problems finding the products that you would like to use?
    2. Have you had issues accessing menstrual products during the COVID-19 pandemic?
21. Have you ever used menstrual products more time than recommended because you could not access another menstrual product?
22. Have you ever used menstrual products more time than recommended because you could not access facilities to change?
23. [Show photography 2, Mexican congresswomen]
    1. Could you describe this photograph?
    2. What do you feel looking at it?
24. Have you noticed any changes in your menstrual cycle or menstruation since the start of the COVID-19 pandemic?
    1. If so, what changes have you experienced?
    2. If so, how have you managed them?
    3. If so, have you sought professional help? How has your experience been accessing healthcare services?
25. Have you had COVID-19 (diagnosed/suspected)?
    1. If so, have you experienced symptoms for over 4 weeks (long COVID)?
    2. Have you noticed any changes in your menstrual cycle or menstruation since you have had COVID-19?

Material S3. Photographs used for the photoelicitation interviews


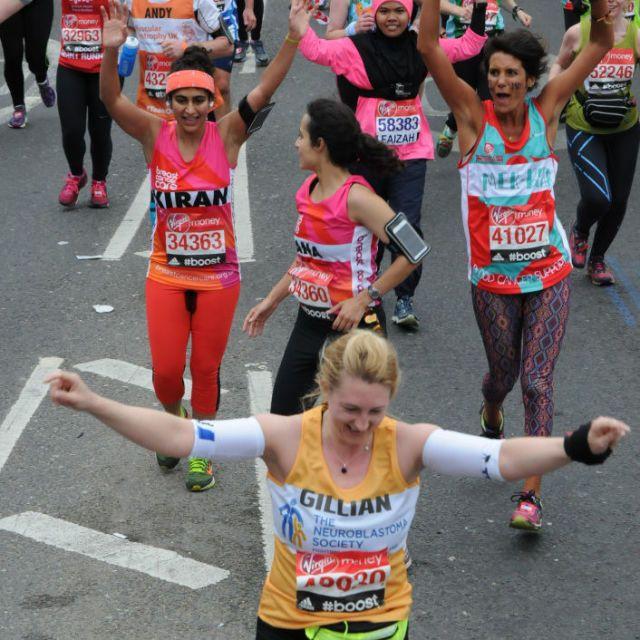


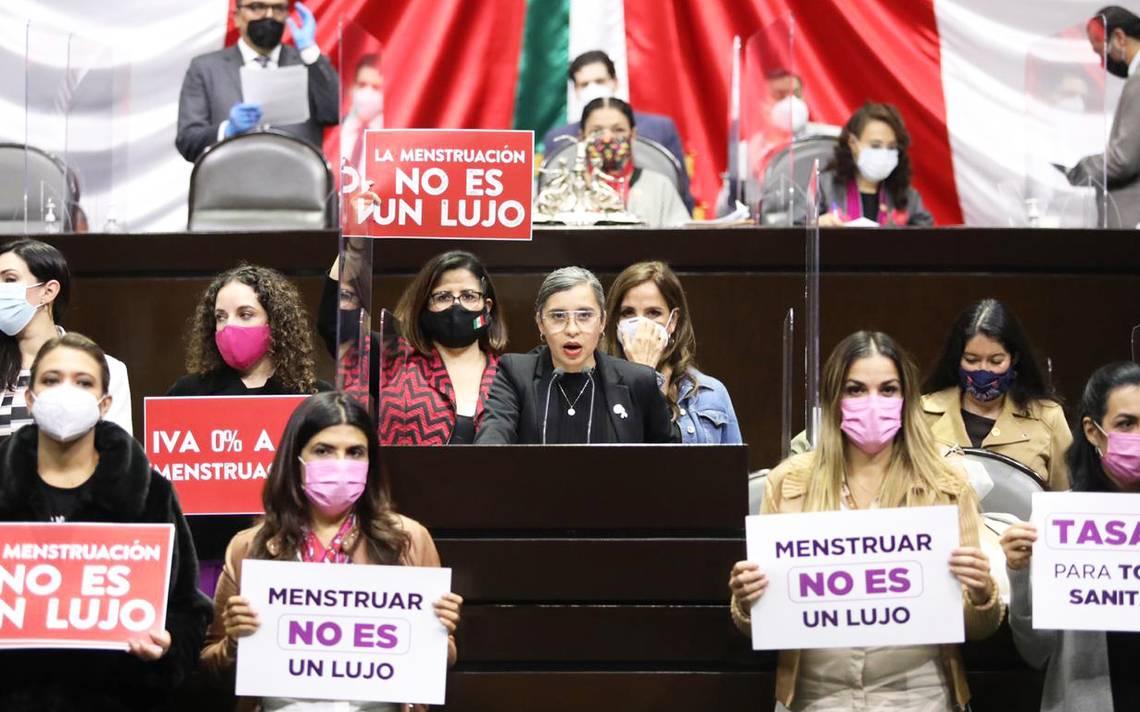
Figure S1. Marathon runners. Kiran Gandhi in the London Marathon with her friends. Photograph from: <https://madamegandhi.blog/2018/12/21/madame-gandhi-featured-in-allures-100-years-of-period-campaign/>

Figure S2. Mexican congresswomen and Menstruación Digna México (@dignamx) representatives. Image from: [*www.elcafediario.com*](http://www.elcafediario.com)
